# The efficacy and safety of multiple versus single doses dexamethasone in unicompartmental knee arthroplasty: A protocol of randomized controlled trial

**DOI:** 10.1097/MD.0000000000021671

**Published:** 2020-08-21

**Authors:** Dehong Gao, Xin Liu, Fan Zhang, Mingyan Ding

**Affiliations:** a Department of Anesthesia, Hanchuan People's Hospital; b Department of Anesthesia, Renmin Hospital of Wuhan University/Hubei General Hospital, Hubei, China.

**Keywords:** complications, dexamethasone, protocol, unicompartmental knee arthroplasty

## Abstract

**Background:**

Concerns exist regarding the analgesia effect and safety of multiple versus single doses dexamethasone in unicompartmental knee arthroplasty. There is an urgent need of studies that efficiently control for confounding, conduct comprehensive and consecutive observation of potential risks of the dexamethasone administration, and investigate its clinical applicability. We thus further designed a randomized controlled study to assess the different dose of dexamethasone on postoperative pain and complications in patients undergoing unicompartmental knee arthroplasty.

**Methods:**

This randomized, prospective, controlled study was carried out between January 2018 and August 2019. It was approved by the institutional review board in our hospital (HBRM2020013). A total of 80 patients were randomly assigned to each group: the study group (n = 40) and the control group (n = 40). All surgical procedures were performed by a similar orthopedic surgeon. In the study group, patients received intravenously 20 mg dexamethasone (4 mL, Tianjin Kingyork group Co., Ltd., China) just after the anesthesia, and repeated at 24 hours after the surgery. Patients in the control group received intravenously 10 mg dexamethasone solution (2 mL) just after the anesthesia, and repeated at 24 hours after the surgery. CRP, IL-6, VAS pain scores at rest and walking, the VAS scores of nausea, and the incidence of postoperative vomiting and nausea (POVN) were recorded at 24, 48, and 72 hours postoperatively.

**Conclusion:**

We hypothesized that patients receiving multiple doses of dexamethasone was associated with better outcomes compared with patients receiving single dose of dexamethasone.

**Trial registration:**

This study protocol was registered in Research Registry (researchregistry5770).

## 1 Introduction

The end-stage knee osteoarthritis is usually restricted to one compartment, it can thus be treated through the unicompartmental knee arthroplasty (UKA).^[1,2]^ In the wake of the aging of the US population, the number of knee arthroplasty is expected to obviously increase by 2030, with the estimated 3.48 million cases each year.^[3,4]^ Although UKA recovers faster than total knee arthroplasty, with better function and fewer complications, it is related to serious postoperative pain. There are many methods for the treatment of the postoperative pain, containing block of femoral nerve, local infiltration anesthesia, and intravenous opioids as well as intrathecal analgesia,^[5–7]^ the management of pain after the UKA is challenging in orthopedic field.

In order to relieve pain and decrease the postoperative inflammation, the multimodal analgesia is needed.^[8]^ Dexamethasone is a kind of long-acting and effective glucocorticoid, which is extensively used in perioperative period.^[9,10]^ Several articles have demonstrated that the dexamethasone with single dose could effectively decrease the postoperative pain.^[11–13]^ Furthermore, the preoperative single dose dexamethasone is an effective medication to prevent nausea and vomiting. Moeen et al^[14]^ have reported that the dexamethasone in 0.25% bupivacaine solution possessed better analgesic effect than alone utilizing the bupivacaine in the surgery of knee arthroscopic. Although most of the researches on the glucocorticoid in UKA have centered on routes of joint administration, as far as we know, the UKA research of glucocorticoids is limited to very few investigation. Because of the clinical heterogeneity, the optimal dosage of dexamethasone, administration mode, and timing in UKA have not been determined, resulting in significant differences in the clinical results.

Although there is an increasing interest in the dexamethasone application in UKA, the results from these approaches are still relatively small. In view of the lack of evidence, the purpose of this research was to clarify the safety and efficacy of the single dose and multiple dexamethasone in the treatment of the postoperative pain and complications after UKA. We assumed that patients who received the dexamethasone with multiple doses had a better prognosis than the patients who received the dexamethasone with single dose.

## 2 Materials and methods

### 2.1 Study design and patients

This prospective, randomized, and controlled research was conducted from January 2018 to August 2019. This single center, double-blinded, randomized controlled trial is conducted in accordance with the Declaration of Helsinki principles. It was authorized via the Institutional Review Committee in our hospital (HBRM2020013) and then was registered in research registry (researchregistry5770). Each patient received the written informed consent. Patients with a diagnosis of the medial compartment osteoarthritis following primary UKA were eligible for inclusion. UKA revision, history of thrombosis, infection, allergy to dexamethasone, use of the glucocorticoids 3 months before the surgery, drug or alcohol abuse, the serious heart disease history (New York Heart Association > 2), body mass index (BMI) >35 kg m^−2^, and the knee joint buckling <90° were excluded. Eighty patients were divided into 2 groups randomly: they are control group (n = 40) and study group (n = 40), respectively. A table of random numbers hidden in the 1:1 ratio was computer-formed via a nurse. The randomization was blind, it was conducted via nurses in sealed envelopes before operation. And at the same time, dexamethasone was prepared by a senior anesthesiologist. All of the anesthesiologists and nurses were not involved, and in the trial, the surgeons, patients, statisticians, and the researchers were blinded.

### 2.2 Dexamethasone administrations

The patients in study group was given dexamethasone 20 mg (4 mL, Kingyork group Co., Ltd., China) intravenously after anesthesia, and repeated at 24 hours after the surgery. In control group, patients were given solution (2 mL) of 10 mg dexamethasone intravenously immediately after anesthesia, and repeated at 24 hours after the surgery.

### 2.3 Surgery, anesthesia, and postoperative care

All UKAs were implemented by a similar orthopedic surgeon. A minimally invasive approach was followed by the upper pole of the patella to the tibial tuberosity. All the operations were carried out under the condition of general anesthesia. All patients were taken 15 mg/kg of tranexamic acid 10 minutes before the incision. 1.5 g cefuroxime was given intravenously to prevent infection 2 hours before the surgery. All UKAs were performed using the Miller-Galante prosthesis (Zimmer; Warsaw, IN). No drainage tubes and tourniquet use. Prior to the wound closure, the ropivacaine (100 mL) of 0.2% was utilized for the local anesthetic. There was no intravenous patient-controlled analgesia or the nerve block during the perioperative period. Two hours after surgery, transferring the patient to the room of anesthesia recovery, afterward, sent the patient to the inpatient department. A gait rehabilitation program was conducted by a physiotherapist, and the patient began walking with a walker on the first day after surgery. Mechanical thromboprophylaxis combined with the chemoprophylaxis was utilized for the prevention of the venous thromboembolism. As a kind of chemical thromboprophylaxis, all the patients were given the hypodermic injection of heparin with low molecular weight (of 2000 IU and 0.2 mL) 6 hours after surgery, and a full dose of (4000 IU, 0.4 mL) was used at 24 hours intervals after the surgery. After discharge, the patient was given oral administration of 10 mg rivaloxaban to prevent the thrombosis for 15 days. As a mechanical thromboprophylaxis, intermittent pneumatic pressure devices were routinely used before walking.

All the patients were treated with the same strategy of pain. Oral analgesic medication 75 mg of pregabalin q8h and 200 mg of celecoxib q12h were utilized to perform the preemptive analgesia 24 hours before operation. At the end of the operation, visual analogue scale (VAS, 10—worst imaginable pain and 0—no pain) was utilized to evaluate the degree of pain. The routine analgesia regimen is the same as before surgery if the VAS level is less than. Oral 10 mg of oxycodone q8h was applied if the level of VAS is vary from 4 to 6. If the patient's level of pain exceeds 6, the 100 mg of pethidine hydrochloride is injected intramuscularly. The nausea was evaluated through utilizing the VAS (VAS, 10—the most severe nausea imaginable and 0—no nausea). When PONV was >2 times or the level of VAS was >4, the 10 mg of metoclopramide was used as the first-line rescue of antiemetic. If still appears the nausea half an hour after metoclopramide with 2 doses, the 5 mg of ondansetron is applied as the second-line rescue of antiemetic.

### 2.4 Outcome measurements

C reactive protein (CRP), interleukin-6 (IL-6), visual analogue scale (VAS) pain scores at rest and walking, the VAS scores of nausea, and the incidence of postoperative vomiting and nausea (POVN) were recorded at 24, 48, and 72 hours postoperatively. Nausea is a kind of subjective feeling related to the impulse to vomit consciously. Vomiting refers to the gastric contents forced discharge from mouth. The dose and total number of the postoperative analgesics (pethidine hydrochloride and oxycodone) were recorded after operation. The dosage and total number of the patients needing antiemetic rescue medicines (ondansetron and metoclopramide) also were recorded after the operation. The fatigue was evaluated utilizing the 10-point NRS (NRS, 10—fatigued and 1—fit) before the operation and at discharge time. The range of motion (ROM) was also evaluated via nurses utilizing the goniometer before the operation and at discharge. A 6-point satisfaction rating was assessed at the time of discharge. The complications along with length of stay (LOS) also were recorded in detail.

### 2.5 Statistical analysis

The sample size was determined for the primary endpoint and was calculated using PASS 2011 software (NCSS, LLC, Kaysville, UT). According to the results of our previous study, the postoperative VAS score was 2.16 in the control group. We anticipated a difference of 0.72 in the VAS score. With a power of 0.90 and significance level of 0.05, the required sample size was calculated as 40 in each arm. Considering possible exclusion, we decided to include 50 patients in each group. Statistical analysis was performed by an independent expert, not involved in the study protocol. Continuous variables were calculated as by student *t* test (which were presented as mean ± SD), such as CRP, IL-6, VAS pain scores at rest and walking, VAS scores of nausea, fatigue scores, ROM, and LOS. All statistical analyses were performed using the SPSS ver. 18.0 (SPSS Inc., Chicago, IL).

## 3 Result

The results will be shown in Tables 1 and 2.

**Table 1:**
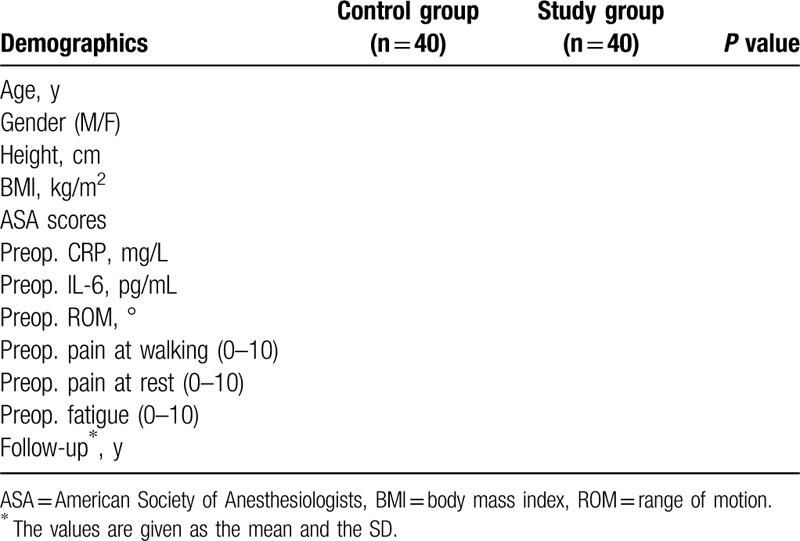
Patient baseline demographics.

**Table 2:**
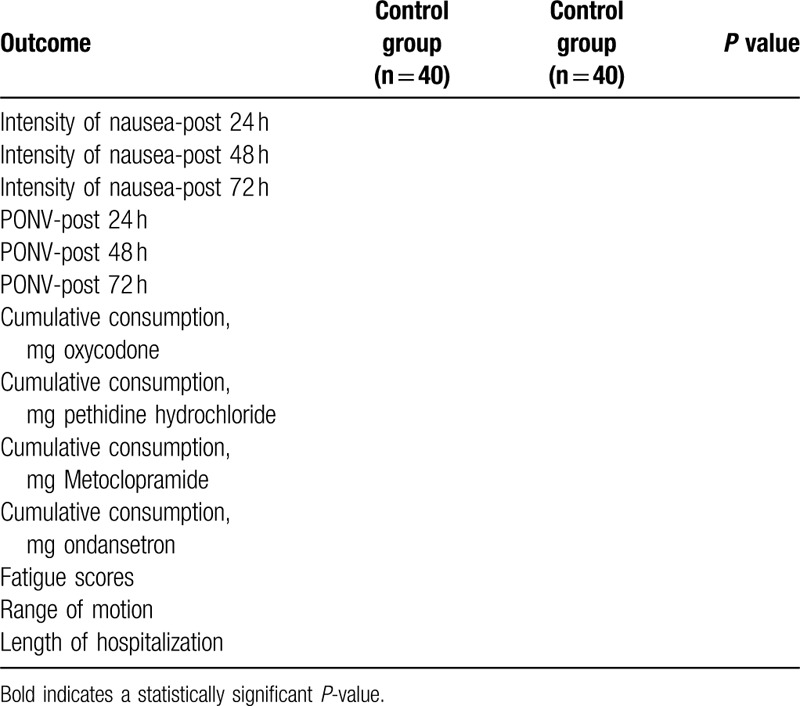
The outcomes in the 2 groups.

## 4 Discussion

Glucocorticoid suppresses the production of prostaglandins by inhibiting Cyclooxygenase-2 mRNA expression centrally and peripherally.^[15,16]^ Furthermore, it inhibits mediators of inflammatory hyperalgesia,^[17]^ such as tumor necrosis factor-a, interleukin-1b, and interleukin-6. These effects require protein synthesis and have a latency of onset. Another late effect of glucocorticoid is suppression of proliferation of intercellular proteoglycans and collagen, thereby reducing adhesion and formation of fibrosis.^[18,19]^ Recently, a shorter onset time for steroid effect has been found. The mechanism of this action is probably transmitted by cell membrane receptors decreasing release of glutamate and g-amino butyric acid.^[20]^ Based on these action mechanisms, the effects of different dose glucocorticoid administration after a UKA have been studied extensively. However, the results have previously been inconclusive. Therefore, we further conducted a double blinded randomized controlled trial (RCT) to assess the efficacy of multiple versus single doses dexamethasone on postoperative pain and complications in patients undergoing UKA. The strength of this study was its prospective and randomized design.

## 5 Conclusion

We hypothesized that patients receiving multiple doses of dexamethasone was associated with better outcomes compared with patients receiving single dose of dexamethasone.

## Author contributions

**Conceptualization:** Dehong Gao.

**Data curation:** Xin Liu.

**Software:** Fan Zhang.

**Study design:** Mingyan Din.
